# Characterisation of the *Cinnamomumparthenoxylon* (Jack) Meisn (Lauraceae) transcriptome using Illumina paired-end sequencing and EST-SSR markers development for population genetics

**DOI:** 10.3897/BDJ.12.e123405

**Published:** 2024-06-17

**Authors:** Mai-Phuong Pham, Dinh Duy Vu, Cui Bei, Thi Tuyet Xuan Bui, Dinh Giap Vu, Syed Noor Muhammad Shah

**Affiliations:** 1 Graduate University of Science and Technology (GUST), Vietnam Academy of Science and Technology, Hanoi, Vietnam Graduate University of Science and Technology (GUST), Vietnam Academy of Science and Technology Hanoi Vietnam; 2 Join Vietnam–Russia Tropical Science and Technology Research Center, Hanoi, Vietnam Join Vietnam–Russia Tropical Science and Technology Research Center Hanoi Vietnam; 3 Jiangsu Vocational Institute of Architectural Technology, School of Architectural Decoration, Xuzhou 221100, Jiangsu, China Jiangsu Vocational Institute of Architectural Technology, School of Architectural Decoration, Xuzhou 221100 Jiangsu China; 4 Institute of Ecology and Biological Resource, Vietnam Academy of Science and Technology, Hanoi, Vietnam Institute of Ecology and Biological Resource, Vietnam Academy of Science and Technology Hanoi Vietnam; 5 Institute of Technology, Hanoi University of Industry (HaUI), Hanoi, Vietnam Institute of Technology, Hanoi University of Industry (HaUI) Hanoi Vietnam; 6 Department of Horticulture, Faculty of Agriculture, Gomal University, Dera Ismail Khan, Pakistan Department of Horticulture, Faculty of Agriculture, Gomal University Dera Ismail Khan Pakistan

**Keywords:** endangered species, genetic diversity, genetic structure, Illumina HiSeq^TM^ 4000, species conservation, SSR markers

## Abstract

*Cinnamomumparthenoxylon* is an endemic and endangered species with significant economic and ecological value in Vietnam. A better understanding of the genetic architecture of the species will be useful when planning management and conservation. We aimed to characterize the transcriptome of *C.parthenoxylon*, develop novel molecular markers, and assess the genetic variability of the species. First, transcriptome sequencing of five trees (*C.parthenoxylon*) based on root, leaf, and stem tissues was performed for functional annotation analysis and development of novel molecular markers. The transcriptomes of *C.parthenoxylon* were analyzed via an Illumina HiSeq^TM^ 4000 sequencing system. A total of 27,363,199 bases were generated for *C.parthenoxylon*. De novo assembly indicated that a total of 160,435 unigenes were generated (average length = 548.954 bp). The 51,691 unigenes were compared against different databases, i.e. *COG, GO, KEGG, KOG, Pfam, Swiss-Prot*, and *NR* for functional annotation. Furthermore, a total of 12,849 EST-SSRs were identified. Of the 134 primer pairs, 54 were randomly selected for testing, with 15 successfully amplified across nine populations of *C.parthenoxylon*. We uncovered medium levels of genetic diversity (PIC = 0.52, Na = 3.29, Ne = 2.18, P = 94.07%, H*o* = 0.56 and He = 0.47) within the studied populations. The molecular variance was 10% among populations and low genetic differentiation (Fst = 0.06) indicated low gene flow (Nm = 2.16). A reduction in the population size of *C.parthenoxylon* was detected using BOTTLENECK (VP population). The structure analysis suggested two optimal genetic clusters related to gene flow among the populations. Analysis of molecular variance (AMOVA) revealed higher genetic variation within populations (90%) than among populations (10%). The UPGMA approach and DAPC divided the nine populations into three main clusters. Our findings revealed a significant fraction of the transcriptome sequences and these newlydeveloped novel EST-SSR markers are a very efficient tool for germplasm evaluation, genetic diversity and molecular marker-assisted selection in *C.parthenoxylon*. This study provides comprehensive genetic resources for the breeding and conservation of different varieties of *C.parthenoxylon*.

## Introduction

*Cinnamomumparthenoxylon* (Jack) Meisn. (Lauraceae) is an evergreen, broad-leaved, and diploid (2n = 24) tree species restricted to Vietnam, India, and China (MOST and VAST 2007). The species is found at elevations above 200 m in the secondary forests of Vietnam in deep, well-drained soil developed from limestone, granite, and alluvial sediment (Nguyen 2010). *Cinnamomum* species are recalcitrant to natural regeneration. Older trees are light-demanding, but young trees may tolerate shade. The tree can reach up to 45 m in height and 85 cm in diameter at breast height. They are a natural source of high-quality essential oil and timber. In addition, *Cinnamomum* is an economically important tree in Vietnam. The excessive use/exploitation of the species by the timber and pharmaceutical industries in recent decades has caused a sharp population decline in *C.parthenoxylon*. The population decline has been exacerbated by the poor regeneration potential and slow growth of the species (Hai et al. 2016). For these reasons, the species has been classified as endangered (CR A1a, c, d) in the Vietnam Red Data Book (MOST and VAST 2007). Human activities in tropical forests mostly reduce the distribution area and increase isolation for many plant species (Yan et al. 2024), which affect genetic variation, gene flow between populations and resistance to environmental stochasticity (Liu et al. 2013, Sebbenn et al. 2017). Genetic diversity in a species contributes greatly to the adaptation of the species to environmental changes for long-term survival (Reed 2002). The genetic diversity within and between populations is a potential evolutionary trait that can lead to the formulation of strategies for species conservation and restoration (Hamrick and Godt 1996).

The past two decades have witnessed extensive applications of molecular techniques to analyze genetic diversity within and among populations of threatened species of Lauraceae, for instance, RAPD and SRAP markers to assess genetic diversity in Sri Lankan *Cinnamomum* species (Abeysinghe et al. 2014), RAPD in *Cinnamomumzeylanicum* Blume (Sandigawad and Patil 2013), EST-SSRs developed on the basis of next-generation sequencing technology in *Neolitseasericea* (Blume.) Koidz (Chen et al. 2015), SSRs developed in *Cinnamomumcamphora* (L.) Siebold (Kameyama et al. 2012, Kameyama et al. 2017) and SSRs in *Cinnamomumbalansae* Lecomte (Cui et al. 2022). However, the genetic diversity and genetic structure of *C.parthenoxylon* have not been explored, which are important prerequisites for formulating and implementing conservation and restoration measures.

Molecular markers are used to assess the genetic diversity and genetic structure of rare and threatened species populations. Microsatellites (SSRs; simple sequence repeats) are highly polymorphic, widely distributed across genomes and highly reproducible, which make them a powerful means to characterise the genetic make-up of plant populations. SSRs developed from expressed sequence tags (EST-SSRs) are gene-based markers (Sahu et al. 2012), with increased identification potential for polymorphisms in woody species (Kaur et al. 2012). Transcriptome sequencing is a simple and effective method for developing numerous SSRs for a comprehensive analysis of the plant genome (Wang et al. 2010, Yan et al. 2015, Hu et al. 2016). Recently, ESTs have become available for a large number of plant species; however, there are currently no ESTs for *C.balansae* in GenBank. Studies on *C.parthenoxylon* in Vietnam have focused on the morphological and ecological characteristics of the species (Tai and Thin 2014, Hai et al. 2016). This study focuses on the transcriptome characterisation, functional analysis, classification and metabolic pathway identification of *C.parthenoxylon* using the Illumina HiSeq^TM^ 4000 platform. A set of EST-SSR markers is developed and used to assess the genetic diversity and genetic structure of the endangered *C.parthenoxylon* population.

## Material and methods

### Plant material

**Figure 1.**
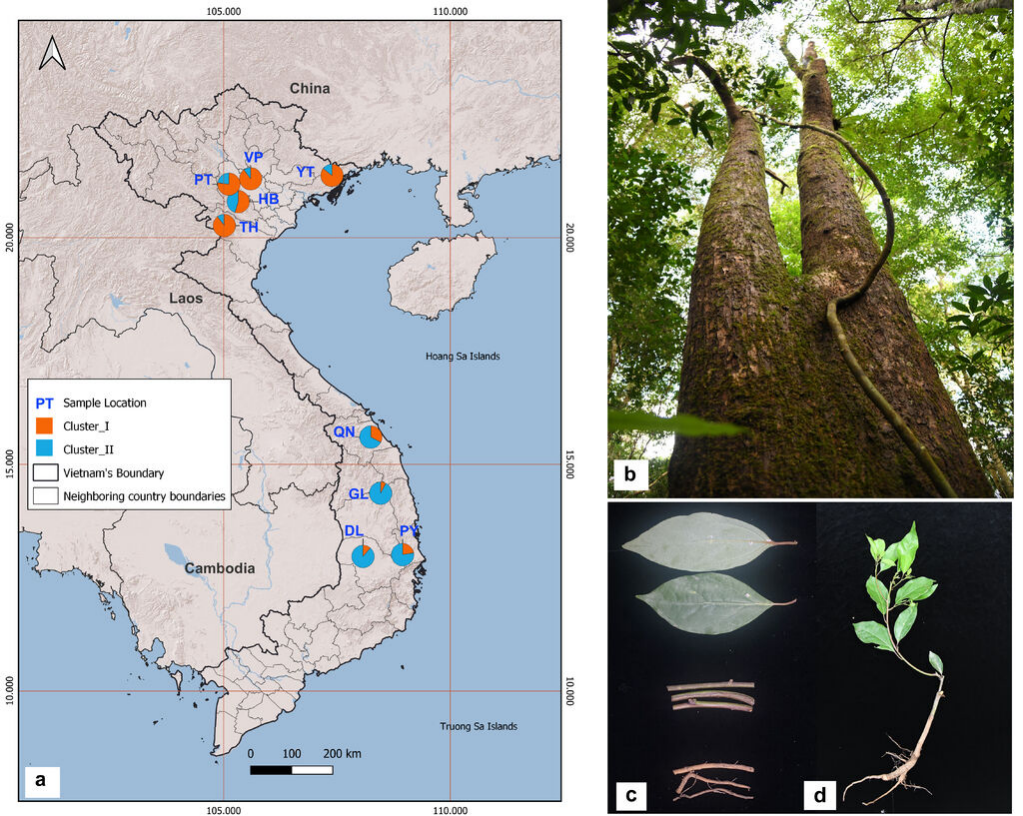
Sampling locations and individuals of *C.parthenoxylon* used for this study in Vietnam. Map showing the collection sites (a), adult plant (b), leaves, stem, roots (c) and sampling plant (d). Different symbols in (a) show genetic clustering into two clusters, as revealed in population structure analyses based on microsatellite data.

To identify the characteristics of the comprehensive transcriptome of *C.parthenoxylon* and to develop a large number of expressed sequence tag-SSR (EST-SSR) markers, root, leaf, and stem tissue (Fig. 1) were collected from five *C.parthenoxylon* trees in a wild population in Tam Dao National Park, Vinh Phuc Province, Vietnam. All samples were immediately frozen individually in liquid nitrogen and stored at -80°C until RNA extraction.

To quantify genetic variation within and among the populations, young leaves were sampled from a total of 179 trees in nine wild populations of *C.parthenoxylon* (Fig. 1, Table 1). The samples were immediately placed in liquid nitrogen upon collection and stored at -30°C.

### RNA isolation and transcriptome sequencing

Total RNA was extracted from the samples using a plant RNA kit (Omega Bioteck Inc.) following the manufacturer’s instructions and treated with DNase-I after extraction. The integrity of the RNA samples was evaluated by gel electrophoresis with 1.2% agarose gel and an Agilent 2100 Bioanalyzer (Agilent Technologies, CA, USA) and the purity was analyzed using a NanodropND-2000 spectrophotometer (NanoDrop Technologies, USA). Total mRNA was purified using oligo magnetic beads. The cDNA libraries were constructed following the manufacturer’s instructions. The Illumina HiSeq^TM^ 4000 platform was used to sequence the transcriptome of *C.parthenoxylon* (Beijing Nuoheyuan Technology Co., Ltd.).

The quality of the raw reads was checked by Trimmomatic v3.0 (Bolger et al. 2014). The reads that had more than 15% low-quality bases, were shorter than 36 bp or were contaminated with adaptors were excluded. Trinity v2.4 (Grabherr et al. 2011) with the default parameters (K-mers = 25) was used for transcriptome assembly. Contigs without ambiguous bases were obtained by conjoining the K-mers in an unambiguous path. The reads were mapped back to contigs by Trinity to build unigenes with the paired-end information. The program identified contigs from the same transcript and the distances between these contigs. The contigs were assembled with Trinity and those that did not extend on either end were sequenced. Such sequences are defined as unigenes. Finally, the overlapping unigenes from the libraries were assembled into a continuous sequence by the overlapping ends of different sequences and redundant sequences were removed to acquire nonredundant unigenes with the maximum possible length by the TIGR Gene Indices clustering tool (TGICL) v2.1 (Pertea et al. 2003).

### Functional annotation analysis

To determine predicted functions, all unigenes were used and compared with the NCBI non-redundant (NR) protein sequences (Deng et al. 2006), Swiss-Prot (Apweiler et al. 2004), Gene Ontology (GO) (Ashburner et al. 2000), Clusters of Orthologous Groups (COG) (Tatusov et al. 2000), Eukaryotic Orthologous Groups (KOG) of proteins (Koonin et al. 2004) and Kyoto Encyclopedia of Genes and Genomes (KEGG) (Kanehisa et al. 2004) databases using the BLAST (E-value 10^-5^) program. HMMER software was used to determine protein sequence similarities with the protein family database (Pfam) (Finn 2005).

### Detection of SSR loci and development of SSR primers

The DNA sequence of each nonredundant gene was obtained by sequencing. All unique sequences were used to determine the composition, frequency, and distribution of SSRs in MISA v1.0. For the search criteria of MISA, the minimum number of repetitions of each corresponding unit size was defined as follows: 1~10, 2~6, 3~5, 4~5, 5~5, and 6~5 (where, for example, 1~10 refers to a single nucleotide as the repeat unit with at least 10 repeats and 2~6 refers to a dinucleotide as the repeat unit with at least 6 repeats). ESTs containing SSRs were deposited in GenBank. SSR primers were designed using Primer v5.0 software. The major parameters for primer design were as follows: primer length (18-24 bp), with an optimum of 20 bp, PCR product sizes of 100-300 bp, annealing temperature between 55 and 65°C with an optimum of 60°C and a GC content of 40-65%, with 50% being optimal. The SSR primer pairs were synthesized by Breeding Biotechnologies Co., Ltd.

### DNA extraction and the identification of SSR polymorphism

Total genomic DNA was extracted from fresh leaves by a DNA extraction kit (Norgenbiotek, Canada). An MM 400 mixer (Retsch) was used to crush the samples in liquid nitrogen. The total DNA purity and integrity were measured by a fluorimeter and then the DNA was diluted to 10 ng µl^-1^. A total of 54 primer pairs were randomly selected among 134 SSR primer pairs for PCR identification and 15 were successfully amplified for assessing the nine wild populations of *C.parthenoxylon* (Suppl. material 2). The PCR amplification reaction was performed in a 25 µl volume comprising 2.5 µl of template DNA, 12.5 µl of 2X Taq Master mix, 1 µl of each primer and 8 µl of deionized water. The settings for running the PCR were as follows: initial denaturation at 94°C for 3 minutes; 40 cycles of 1 minute at 94°C, 30 s at a 54-56°C annealing temperature for primer pairs, 1 minute of extension at 72°C and 10 minutes at 72°C for the final cycle; and holding at 4°C. The PCR products were sized and subjected to relative quantification between samples on a 5300 Fragment Analyzer (Agilent) with an Agilent DNF-905 dsDNA Kit (1-500 bp) (Agilent).

### Data analysis

#### Genetic diversity

Null alleles and other genotyping errors were detected using MICRO-CHECKER v.2.0 software (Van Oosterhout et al. 2004), with 1000 bootstrap iterations over loci to generate the expected homozygote and heterozygote frequencies. Deviations from Hardy-Weinberg Equilibrium (HWE) for loci within populations were tested in Cervus (Kalinowski et al. 2007) based on 1000 permutations of alleles among individuals. CERCUS (Kalinowski et al. 2007) was used to estimate each locus' PIC (polymorphism information content) value. Variables representing genetic diversity per locus and population, including the number of alleles (*Na*), number of effective alleles (*Ne*), observed heterozygosity (*Ho*), expected heterozygosity (*He*), inbreeding coefficient (*Fis*) and gene flow (*Nm*), calculated using the program GenAlEx v.6.5 (Peakall and Smouse 2010). The individual inbreeding model was performed to evaluate the *Fis* index for null allele frequency F_IS_IIM using INEst (Chybicki and Burczyk 2009). BOTTLENECK v.1.2 was used to examine each population for deviations in heterozygosity from the expected level for that population given mutation-drift equilibrium (Piry et al. 1999). Two different mutational and drift models, SMM (the stepwise mutation model) and TPM (the two-phased model of mutation) were considered. For TPM, 70% single-step mutation and 10,000 repeats were used. A one-tailed Wilcoxon signed rank test was utilised to determine the significance of the findings.

#### Genetic differentiation and population structure

The *F_ST_* (Weir and Cockerham 1984) and *G'_ST_* values (Hedrick 2005) were used to quantify the genetic divergence between the populations (GenAlEx v.6.5). Using ARLEQUIN v.3.1, the significance of the F_ST_ values for population pairs was assessed at a significance level of 0.05. The results of this testing were considered significant (Excoffier et al. 2005). Analysis of molecular variance (AMOVA) was carried out with the assistance of ARLEQUIN v.3.1, which was used to perform significance testing for the variance components involved. The outcomes of 10,000 different permutations served as the basis for this testing. The genetic association among populations was determined by Poptree2 using the UPGMA approach (Takezaki et al. 2010). Analysis of population structure was performed using STRUCTURE v.2.3.4 and the Bayesian clustering method was used (Pritchard et al. 2000). Establishing the admixture model with correlated allele frequencies required ten distinct runs for each number of groups in the dataset (*K*). These runs were carried out with 500,000 Markov Chain Monte Carlo (MCMC) iterations and a burn-in time of 100,000 iterations. *K* ranged from 1 to 15. STRUCTURE HARVESTER (Earl and Vonholdt 2012) was used to detect the number of groups that best fit the dataset based on the K that was determined by Evanno et al. (2005) to determine the ideal value of *K*. This was done so that the optimal value of K could be determined. When the optimal K value had been determined, the duplicated findings were aligned with CLUMPP v1.1.2 (Jakobsson and Rosenberg 2007) and the allocated cluster membership bar plots were created with DISTRUCT v.1.1. (Rosenberg 2004). A discriminant analysis of principal components (DAPC) was also carried out by utilising the adegenet package in the R v4.0.2 program to discover clusters of individuals with a common genetic ancestor (Jombart et al. 2010). The DAPC was implemented without any previous information on the population's origin. The Bayesian information criterion was used to establish the "ideal" cluster size and the number of clusters, denoted by the letter "*K*," ranged from one to twenty in the experiments (BIC). The DAPC was also responsible for performing the previous information analysis to determine how individuals should be assigned to populations. To obtain a better visual representation of the genetic clusters, the complot function of adegenet was applied. The *xval*DAPC function was used to retain the top fourteen principal components from the principal component analysis (explaining 98.5% of the variance), as well as the seven discriminant eigenvalues.

## Results

### Illumina sequencing and de novo assembly of transcriptome

*Cinnamomumparthenoxylon* transcriptome sequencing produced 27,363,199 high-quality and clean paired-end reads with a total output of 8.17 Gb and 160,435 unigenes (235 Mb) were assembled. The GC content was 46.2%, the Q20 score was 99.15% with 100% cycles and the Q30 score was 97.05%. Trinity produced 3,274,271 contigs with a mean length of 78.66 bp and an N50 of 110 bp. In total, 3,167,763 (96.75%) contigs were between 200 and 300 bp in length; 53,394 (1.63%) ranged from 301-500 bp; 32,240 (0.98%) ranged from 501-1000 bp; 14,348 (0.44%) ranged from 1001-2000 bp; and 6,526 (0.2%) transcripts were longer than 2000 bp (Suppl. material 3, Table 2). Similarly, 262,999 transcripts were produced with a mean length of 902.49 bp and an N50 of 1682 bp. Among the transcripts, 81,432 (30.96%) transcripts ranged between 200 and 300 bp in length; 58,136 (22.11%) transcripts ranged from 301 to 500 bp; 47,630 (18.11%) transcripts ranged from 501-1000 bp; 43,206 (16.43%) transcripts ranged from 1001-2000 bp; and 32,595 (12.39%) transcripts were longer than 2000 bp (Suppl. material 4, Table 2). A total of 160,435 unigenes were produced, with a mean length of 548.95 bp and an N50 of 711 bp. Among these unigenes, 71,571 (44.61%) were between 200 and 300 bp in length; 44,960 (28.02%) were 301 to 500 bp; 25,658 (15.99%) were 501 to 1000 bp; 11,776 (7.34%) were 1001 to 2000 bp; and 6,470 (4.03%) had a length of more than 2,001 bp (Suppl. material 5, Table 2).

### Functional annotation and classification of unigenes

**Figure 2.**
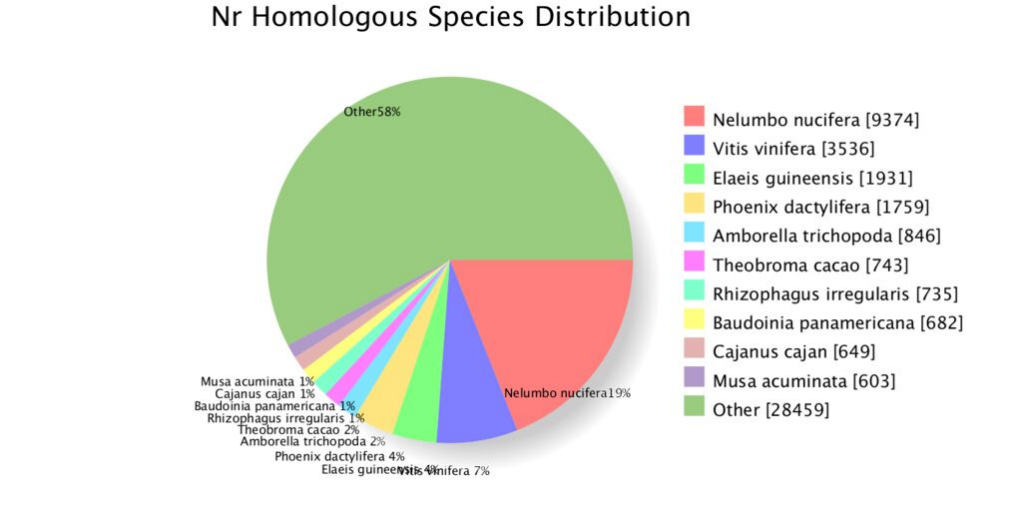
Distribution of species search of unigenes against the Nr database.

A total of 51,691 sequences among the blasted unigenes had matches in the public databases, i.e. *COG, GO, KEGG, KOG, Pfam, Swiss-Prot* and *NR* (Table 3). Out of 160,435 unigenes, 14,629 (9.12%) unigenes were annotated against the COG database, 10,701 (6.67%) unigenes against the KEGG database, 26,823 (16.72%) unigenes against the KOG database, 29,514 (18.39%) unigenes against the *Pfam* database, 17,436 (42.35%) unigenes against the *Swiss-Prot* database and 25,352 (15.8%) unigenes against the GO database (Table 3). The similarity distributions of *C.parthenoxylon* with different species were derived using BLASTx against the Nr database. The *C.parthenoxylon* genes shared 19% similarity with those of *Nelumbonucifera* Gaertn., 7% with those of *Vitisvinifera* L., 4% each with those of *Elaeisguineensis* L. and *Phoenixdactylifera* L. and 66% with those of other species (Fig. 2).

**Figure 3.**
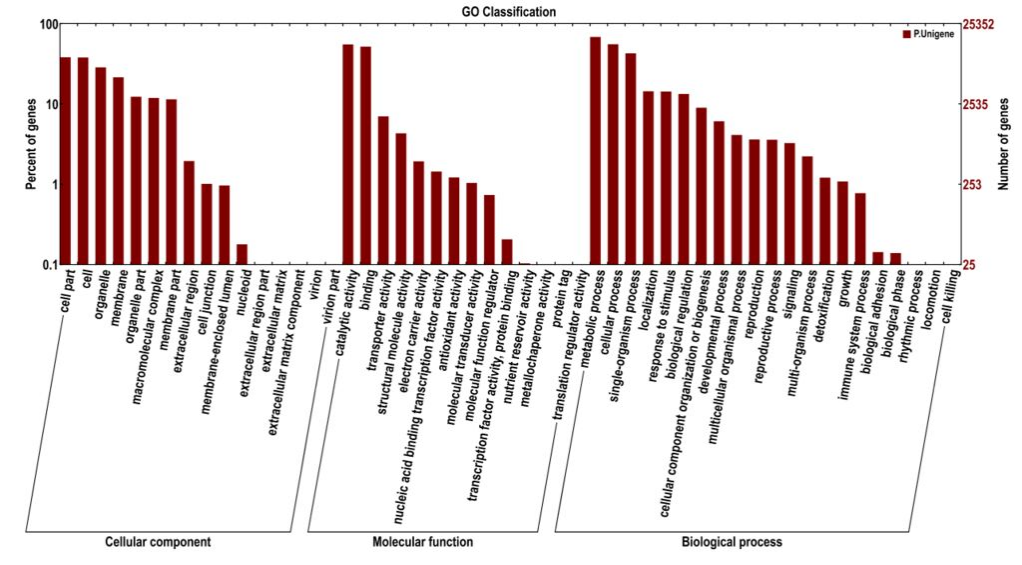
Gene Ontology (GO) classifications of unigenes of *C.parthenoxylon*. A total of 25,352 unigenes were categoried into three main categories: biological process, cellular component and molecular function. The x-axis indicates the subgroups in GO annotation, while the y-axis indicates the percentage of speciﬁc categories of genes in each main category.

**Figure 4.**
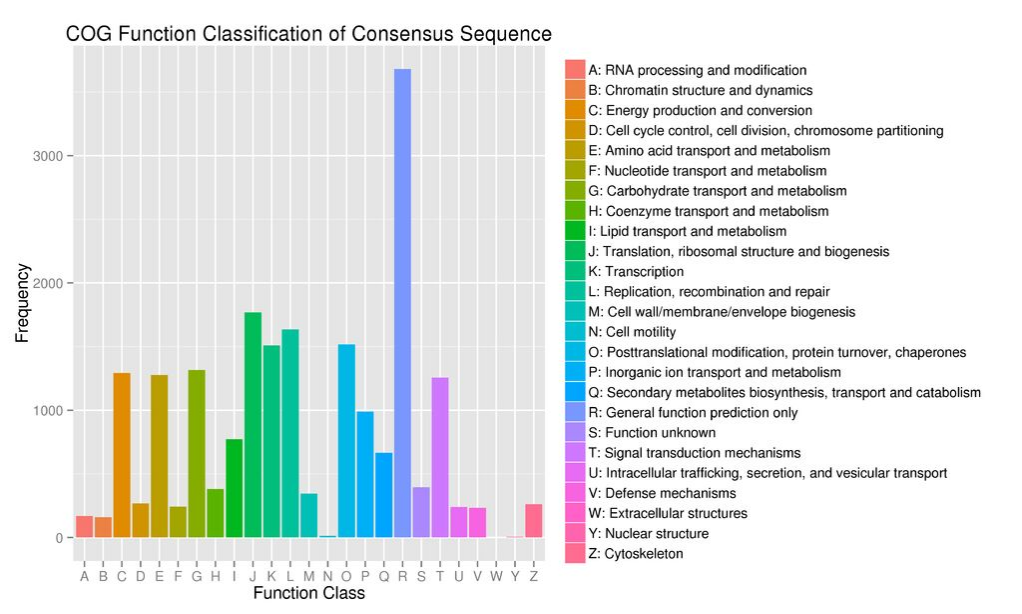
Classification of the COG by functional categories of *C.parthenoxylon*. One-letter abbreviations for the functional categories (A-Z).

**Figure 5.**
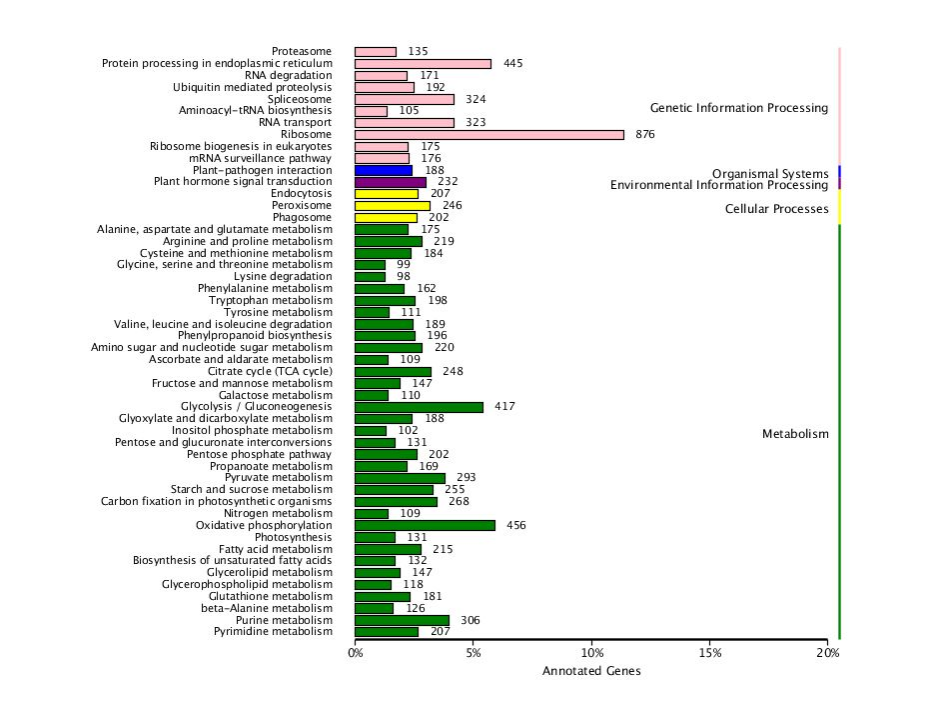
Clusters of orthologous groups based on KEGG classification of *C.parthenoxylon* (Cellular process; Environmental information processing; Genetic information processing; Metabolism; Organismal systems).

A total of 25,352 (15.8%) unigenes were assigned GO annotations and could be divided into three ontologies (biological process, molecular function and cellular component) with 51 subcategories (Fig. 3). The biological process was associated with the largest number of unigenes (43.11%), followed by "metabolic process", "cellular process" and "single-organism process", which were associated with 17,389 (38.85%), 14,108 (31.52%) and 10,864 (24.27%) unigenes, respectively. In the molecular function (37.81%) category, genes encoding "binding" (13,167; 41.44%) proteins and proteins related to "catalytic activity" (14,034; 44.18%) were prominently represented. Moreover, some genes were also recorded for "metallochaperone activity", "nutrient reservoir activity", "protein tag" and "translation regulator activity". The cellular component category represented 19.07% of unigenes, among which 9,645 (48.7%) unigenes were associated with "cell" and 9,708 (49.02%) unigenes were associated with "cell part", while the other major subcategories included "organelle", "membrane", "organelle part", "macromolecular complex" and "membrane part". A total of 14,629 (9.12%) unigenes were assigned to 25 COG categories (Fig. 4). “General function prediction only" was the primary group (9,089), followed by "translation, ribosomal structure and biogenesis" (3,388) and "replication, recombination and repair" (977). Few unigenes represented "extracellular structures" and "nuclear structure". The unigenes were subjected to KEGG pathway analysis (E<E^-5^). A total of 10,701 (6.67%) unigenes had significant matches in the KEGG pathway databases and were matched to 119 KEGG functional pathways (Fig. 5). The primary pathways included ribosome, RNA transport, spliceosome, purine metabolism, plant hormone signal transduction and others.

### Frequency, distribution and characterisation of SSRs from the C.parthenoxylon transcriptome

**Figure 6.**
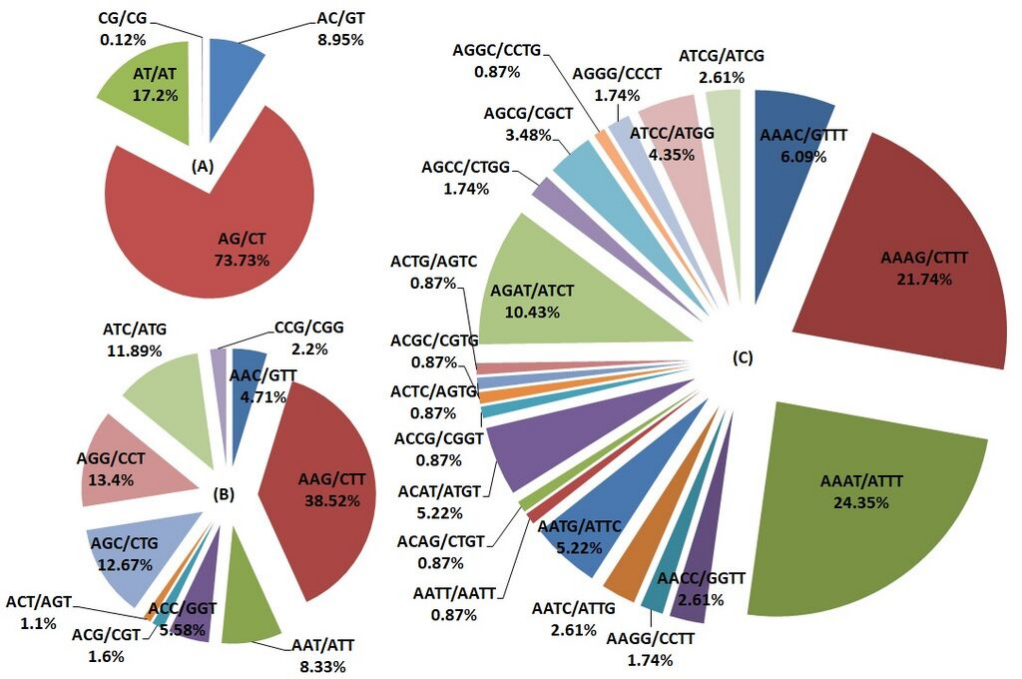
Percentage of diﬀerent motifs in di-nucleotide (A), tri-nucleotide (B), and tetra-nucleotide (C) repeats in *C.parthenoxylon*.

For the development of new molecular markers, the 160,435 assembled unigenes were used to explore the potential microsatellites, which were defined as di- to hexanucleotide motifs. A total of 12,849 potential EST-SSRs were identified by the SSRIT tool. A total of 8,687 and 2,917 sequences had one and more than one microsatellite locus, respectively, while the EST-SSR frequency was 7.5% and the distribution density of one EST-SSR was 4.16 kilobases (kb) among the unigenes. The most common repeat motif was mononucleotide (7,183; 55.90%), followed by dinucleotide (3,331; 25.92%), trinucleotide (2,186; 17.01%), tetranucleotide (115; 0.9%), hexanucleotide (17; 0.13%) and pentanucleotide (17; 0.13%) repeats (Table 4, Suppl. material 6). Ten repeat motifs (2,496; 19.43%) was the most frequent number among the EST-SSRs, followed by six tandem repeats (1,508; 11.74%), five tandem repeats (1,404; 10.93%), seven tandem repeats (862; 6.71%), eight tandem repeats (609; 4.45%) and nine tandem repeats (700; 5.45%). Among the dinucleotide repeats, the dominant nucleotide repeat was AG/CT (2,456), followed by AT/TA (573), AC/TG (298), and CG/GC (4). Among the trinucleotide repeats, AAG/CTT (842) was the most common, followed by AGG/CCT (293), AGC/CTG (277), ATC/ATG (260), AAT/ATT (182), ACC/GGT (122), AAC/GTT (103), CCG/CGG (48), ACG/CGT (35) and ACT/AGT (24). The most common motif types of tetranucleotide repeats were AAAT/TTTA (28) and AAAG/CTTT (25) (Fig. 6, Suppl. material 1). Additionally, 15 different types of pentanucleotide repeat EST-SSRs and 22 different types of hexanucleotide repeat EST-SSRs were also identified.

### Population genetic diversity and structure

A total of 54 primer pairs were randomly selected among 134 SSR primer pairs for PCR identification and 15 were successfully amplified for the study of the wild population of *C.parthenoxylon* at nine different locations (Suppl. material 2). Null allele frequencies were determined at four loci (VDD07, VDD08, VDD11 and VDD14) for *C.parthenoxylon* at a significance level of 0.05. Ten of the 15 loci displayed heterozygote deficits under the Hardy–Weinberg Equilibrium in *C.parthenoxylon* (Table 5). We found 97 different alleles of 104 to 260 bp at the 15 loci from the 179 sampled trees of nine *C.parthenoxylon* populations. Table 4 shows that Na = 3.29 (2.00-4.89), Ne = 2.18 (1.24–3.11), PIC = 0.52 (0.18-0.77), H*o* = 0.56 (0.08–0.86) and He = 0.47 (0.14-0.66). The fixation index (F_IS_) was -0.145. Positive *Fis* values were found at all four loci (VDD07, VDD08, VDD11 and VDD14) under investigation, suggesting an excessive number of homozygotes and inbreeding. The inbreeding coefficient of the populations (*Fit* = -0.002) suggests an excess of heterozygotes in the populations (Table 5).

Genetic diversity was recorded at the population level (Table 1) and the data showed 3.29 mean alleles per locus (*Na*) and 2.18 mean effective alleles (*Ne*). The proportion of polymorphic loci was 94.07%, where *Ho* and *He* were 0.56 and 0.47, respectively. The values of the inbreeding coefficient (*Fis*) were negative for the six populations (QN, VP, HB, PY, YT and PT), which proved that these populations are predominantly allogamous, further suggesting outcrossing and reflecting the population structure. The values of *Fis* were positive for three populations (DL, GL and TH), suggesting a departure from Hardy–Weinberg quilibrium with excess homozygosity. The F_IS_IIM values varied from 0.015 (PY) to 0.038 (HB), with an average of 0.02. The bottleneck analysis showed significant heterozygosity deficits in the VP population (p<0.05) under the SMM (stepwise mutation model) and TPM (two-phase model) (Table 1). This suggests that the VP population recently experienced a bottleneck.

Analysis of molecular variance (AMOVA) was performed based on 1000 permutations and showed that the molecular variation was attributable to differentiation within the populations of *C.parthenoxylon* (Table 6). The results revealed that the total variation within populations was 90%, which was highly significant (P<0.001). Genetic variation between the populations was recorded as 0.06 (0.01-0.14) (P < 0.05), indicating moderate genetic differentiation, while gene flow was 2.16. The highest differentiation value (Fst = 0.14) was observed within the YT and GL populations and the lowest (Fst = 0.01) was observed within the PT and VP populations (Table 7).

**Figure 7.**
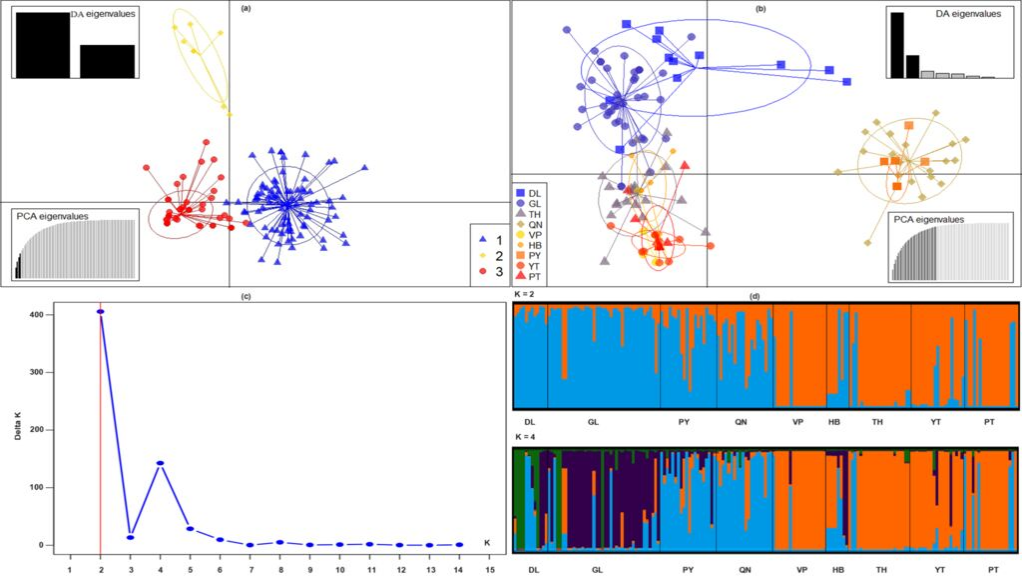
Analysis of population structure using DAPC and STRUCTURE for nine *C.parthenoxylon* populations. Scatterplot of the DAPC with prior information (a), Scatterplot of the DAPC without prior information (b), Distribution of DeltaK over K=1-15 (c) and Bar plots at K = 2 and K = 4 (d).

Without any prior information, discriminant analysis of principal components (DAPC) also uncovered three genetic groupings for *C.parthenoxylon* (Fig. 7b). Cluster 1 included two populations from the Central Highlands area (GL and DL), Cluster 2 included two populations from the South Central Coast area (QN and PY) and Cluster 3 included five populations from the Northwest and Northeast areas (HB, VP, PT, YT, TH and TQ). DAPC performed with prior information on population origin showed individuals within and between populations (Fig. 7a). The high overlap indicated low genetic differentiation between populations.

The Bayesian analysis of individual assignments, based on the likelihoods, showed that the highest ∆K value (405.5) for 179 individuals was associated with K = 2 as the optimum number of genetic groups and showed that all individuals exhibited admixture among the three groups (Fig. 1, Fig. 7c and d). The proportion of ancestry linked with each genetic group can be deduced from the color of each individual, as measured by the percentage of the population's total color. At K = 2, the *C.parthenoxylon* populations were divided into two groups (Central Highlands and South Central Coast; Northwest and Northeast). The percentage of the color of 179 individuals showed the fraction of ancestry associated with each genetic group. The blue group contained four populations (GL, DL, QN and PY), while the orange group included five populations (HB, VP, PT, YT, TH and TQ). At K = 4, the *C.parthenoxylon* populations were diﬀerentiated into five groups.

**Figure 8.**
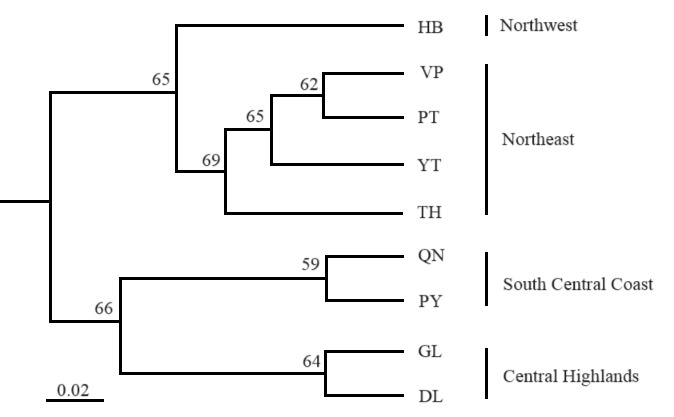
Unweighted pair group method average cluster analysis tree based on Nei’s chord distance of genetic relationship among nine *C.parthenoxylon* populations.

The distinct genetic groups of the populations were identified by the unweighted pair group method average cluster analysis (UPGMA) method based on Nei’s distance by using POPTREE2 (Fig. 8). Three different groups were generated; the first group was composed of six populations from the Northwest and Northeast areas (HB, VP, PT, YT, TH and TQ) with a bootstrap value of 65%, the second group was composed of two populations from the South Central Coast area (QN and PY) with a bootstrap value of 59% and the last group was composed of two populations from the Central Highlands area (GL and DL) with a bootstrap value of 64%. The populations VP/PT, QN/PY and GL/DL had a close relationship with each other, with high bootstrap values of 62%, 59% and 64%, respectively.

## Discussion

In the present study, the first transcriptomic analysis of *C.parthenoxylon* was performed via Illumina HiSeq™ 4000 sequencing technology. Transcriptome sequencing of *C.parthenoxylon* provided a valuable resource for the development of SSR markers to study the genetic diversity, molecular marker-assisted breeding and evolution of the Lauraceae family. After the uni-transcripts were assembled, the GC content (46.2%) of *C.parthenoxylon* was found to be lower than that of *Arundodonax* L. [49%; Evangelistella et al. (2017)], but higher than that of *Boeaclarkeana* Hemsl. [45.43%, Wang et al. (2017)] and *Fraxinusvelutina* (Gamble) N.Chao ex H.W.Li [45.52%; Yan et al. (2017)] determined using a similar methodology. The GC content provides valuable information regarding the stability of genes and genomic composition for the evolution and genetic structure of the species. These results can be attributed to the adaptability of species to different environmental conditions.

To uncover the prevailing genetic diversity and evolution patterns of *C.parthenoxylon*, databases such as COG, GO, KEGG, KOG, Pfam, Swiss-Prot and NR were utilied to investigate matching sequences. *Cinnamomum* parthenoxylon unigenes were annotated among GO categories. The metabolic process term in the biological process category and the cell term in the cellular components category were the largest group in this study, indicating the importance of cellular and metabolic activities. These results are similar to those of previous studies on *Panicummiliaceam* L. (Yue et al. 2016) and IrislacteaPall.var.chinensis (Fisch.) Koidz (Gu et al. 2018). A total of 14,629 (9.12%) unigenes were assigned to 25 COG categories. Among these categories, ''general function prediction only'' and "translation, ribosomal structure and biogenesis'' were dominant. The current COG database results are parallel to those previously reported for *Camelliataliensis*. (W.W.Sm.) Melchio (Zhang et al. 2015) and *Brassicacampestris* L. (Chen et al. 2017); however, the results are in contrast with those for *Rosaroxburghii* Tratt. (Yan et al. 2015), *Calotropisgigantea* L. (Muriira et al. 2015) and *Neottopterisnidus* L. (Jia et al. 2016), suggesting uncovered diversity in *C.parthenoxylon*. Most likely, the assembled unigenes are the major elements involved in different metabolic pathways and molecular functions. For this purpose, KEGG resources were used to study the biological functions and interactions of genes. Some of the dominant pathways were the ribosome (876 members), oxidative phosphorylation (456), protein processing in the endoplasmic reticulum (445), glycolysis/gluconeogenesis (417), spliceosome (324), RNA transport (323) and purine metabolism (306).

Illumina sequencing technology is an efficient method for obtaining large amounts of transcriptome data for the identification of novel genes and the development of molecular markers. The current study suggested that the transcriptome sequencing of *C.parthenoxylon* provided a valuable resource for the development of EST-SSR markers to study genetic diversity, molecular marker-assisted breeding and evolution in the Lauraceae family. A total of 160,435 transcriptome unigenes were obtained using the Illumina HiSeq^1M^ 4000 platform for *C.parthenoxylon*, with an N50 of 711 bp and a mean length of 548.95 bp. *Cinnamomumparthenoxylon* has shorter unigenes than *Cinnamomumlongepaniculatum* (Gamble) N.Chao ex H.W.Li (N50 = 1387 bp; average length = 879.43 bp) (Yan et al. 2017), *C.camphora* (N50 = 1430 bp; average length = 997 bp) (Li et al. 2018) and *N.sericea* (N50 = 1200 bp; average length = 733 bp) (Chen et al. 2017). A total of 12,849 SSRs were identified from the *C.parthenoxylon* transcriptome analysis. The number of SSRs obtained from transcriptome analysis of *C.parthenoxylon* (12,849 SSRs) was lower than those reported in *N.sericea* (13,213 EST-SSRs) (Chen et al. 2017), *Linderaglauca* (Siebold & Zucc.) Blume (20,048 EST-SSRs) (Zhu et al. 2016) and *C.longepaniculatum* (23,463 SSRs) (Yan et al. 2017). The mono- and di-nucleotide repeat motifs were the most abundant repeat types, followed by tri-, tetra-, hexa- and pentanucleotides. These results in *C.parthenoxylon* were in line with those previously reported for *N.sericea* (Chen et al. 2017) and *C.longepaniculatum* (Yan et al. 2017). The A/T motif was most frequent than C/G, accounting for 99.2% of the mono-nucleotide SSR markers. Among the four types of di-nucleotide motifs in *C.parthenoxylon*, AG/CT was recorded at the highest frequency (73.73%). These values are higher than those in *L.glauca* (37.5%) (Zhu et al. 2016), *N.sericea* (30.02%) (Chen et al. 2017) and *C.longepaniculatum* (Yan et al. 2017), where CG/CG repeat motifs were the least frequent (0.12%). CG motifs were very rare in *C.parthenoxylon*, which is similar to the previously reported results in *N.sericea* (Chen et al. 2017) and *C.longepaniculatum* (Yan et al. 2017). The most abundant trinucleotide repeat motifs were AAG/CTT (38.52%). Similar results were also reported in *C.longepaniculatum* (Yan et al. 2017), *L.glauca* (Zhu et al. 2016) and *N.sericea* (Chen et al. 2017). Thus, A/T, AG/CT and AAG/CTT were the major SSR markers in *C.parthenoxylon*. SSRs can be used to analyse differences between the *C.parthenoxylon* genome and the genomes of other plants within the same family, which can reveal genetic diversity and provide a set of genetic markers.

In the present study, the mean PIC value was 0.52 for 15 polymorphic EST-SSR markers, showing a high level of informativeness (Botstein et al. 1980). These markers could contribute to genetic analysis and aid in the accumulation of genetic information on *C.parthenoxylon* origins. In this study of *C.parthenoxylon*, the 15 SSR markers showed a moderate level of genetic diversity (H*o* = 0.56 and H*e* = 0.47). However, high genetic diversity levels were reported in *Machiluspseudokobu* Koidz. (H*e* = 0.47) (Tsuneki et al. 2009), *L.glauca* (He = 0.56) (Zhu et al. 2016), *C.camphora* (H*o* = 0.345-0.45 and H*e* = 0.423-0.44) (Li et al. 2018, Zhong et al. 2019), *N.sericea* (H*e* = 0.51) (Chen et al. 2017) and *Ocotea* species such as *Ocoteacatharinensis* Mez. (He = 0.73), *Ocoteaodorifera* (Vell) Rohwer (He = 0.78) and *Ocoteaporosa* (Nees & Mart) Barroso (H*e* = 0.64) (Martins et al. 2015). Among the targeted populations, three (DL, GL and TH) showed Fis values ranging from 0.01 to 0.14, indicating a deficit of heterozygotes due to inbreeding. These results suggest a small neighbourhood size and matings between siblings within populations. Further results revealed a sign of a bottleneck for the GL population (*P* < 0.005). The heterozygosity deficit detected by bottleneck analysis showed a reduction in the population size of *C.parthenoxylon*. Small populations may be the result of inbreeding, which may reduce genetic diversity. However, bottlenecks were also detected in the VP population, implying that population sizes were rapidly reduced in the 1980s and 1990s (MOST and VAST 2007, Vu et al. 2017, Nguyen et al. 2021), leading to a lack of gene exchange in the population and a sharp decrease in allele frequency.

Differences in the genetic structure of populations are very important for exploring genetic diversity and *Fst* is an effective metric with which to study genetic differentiation and gene flow among populations (Liu et al. 2013, Peng et al. 2017). Genetic differentiation is strongly affected by gene flow and genetic drift (Hamrick and Godt 1996). Among the tested *C.parthenoxylon* populations, low levels of genetic differentiation (*Fst* = 0.06) and gene flow (*Nm* = 2.16) were found. Strong gene flow (Nm > 1) leads to a high number of migrants in each generation and may prevent genetic differentiation among populations due to genetic drift (Slatkin 1985). A barrier to gene flow is reflected by significant differentiation (10%; p<0.001) in *C.parthenoxylon*. Gene flow, genetic drift, selection, mutations and long-term evolution have a strong effect on genetic variations among different populations (Finkeldey and Hattemer 2007, Brutting et al. 2012, Yan et al. 2024). Literature reports lower variations within populations and mating systems and strong gene flow in *C.camphora* (Kameyama et al. 2017, Kameyama et al. 2012, Li et al. 2018) and *N.sericea* (Chen et al. 2017). High genetic exchange in these species, probably via the dispersal of pollen grains and seeds, may contribute to preserving genetic variation by avoiding local gene drift. Pollen grains dispersed through insects might be a key contributor to gene flow (Brutting et al. 2012). As a woody species, *C.parthenoxylon* has large longevity and is predominately outcrossing. Its pollen grains can be dispersed through the wind and insects (bees). Due to the fact that dispersal depends on insects, an increase in geographic distance will decrease the transmission of pollen grains and seeds between populations. Therefore, habitat fragmentation can lead to increasingly isolated populations and then decrease gene exchange among the populations. This can affect the genetic structure of *C.parthenoxylon*. Our study showed low levels of genetic difference in the same areas, such as GL and DL (Fst = 0.07) in Central Highlands, QN and PY (Fst = 0.05) in the South Central Coast VP and PT (Fst = 0.01) in the Northeast area. High genetic differences were recorded between populations located in different areas, such as Fst = 0.14 between GL/VP and GL/YT, Fst = 0.13 between GL/HB and GL/PY, Fst = 0.12 between GL/PT and Fst = 0.11 between DL/PY all of which are separated by large geographic distances. Moreover, the dispersal of seeds through the wind may be restricted due to their heavy weight. In our study, the UPGMA tree and DAPC revealed closer relationships among the *C.parthenoxylon* populations and two different genetic clusters were identified by structure analysis based on the Bayesian model, with a Δ*K* value at K = 2. However, the structural analysis showed that the 179 genotypes of *C.parthenoxylon* were divided into three clusters in the UPGMA tree and DAPC (Central Highlands and South Central Coast; Northwest and Northeast areas of Vietnam). This reflects the existence of genetic structure as a consequence of genetic exchange, regarding the populations in the identified genetic clusters. The Bayesian clustering approach performed by Structure was more suitable than the UPGMA tree based on genetic distances between the populations. To prevent inbreeding depression and increase the variability of progenies through outcrossing in the future, all the targeted populations could be considered for both *in-situ* and *ex-situ* conservation activities. The PY and VP populations maintained high genetic diversity and can be considered high priorities for *in-situ* conservation to promote the sustainable utilisation of genetic resources. All populations should be considered for germplasm collection to maximise genetic diversity.

## Conclusions

In this study, for the first time, Illumina HiSeq™ 4000 sequencing technology was applied for transcriptomic analysis of *C.parthenoxylon* in Vietnam. A total of 12,849 EST-SSRs were recorded and 134 SSR loci were deposited in GenBank (OR536813-OR536946). The present study shows that *C.parthenoxylon* currently maintains medium levels of genetic diversity and shows low genetic differentiation among populations. The EST-SSRs generated in this study for *C.parthenoxylon* will aid in further exploration of allopatric speciation, adaptive divergence in genetic and lineage diversity and the migration history of *C.parthenoxylon*. Our study may also contribute to taxonomic studies as well as evolutionary research. The current study also provides a platform for breeding and conservation of *C.parthenoxylon* as well as related species.

## Supplementary Material

A42951A3-18B9-5D3F-BCCF-636AC1EDD96A 10.3897/BDJ.12.e123405.suppl1 Supplementary material 1 Table S1. Frequency distribution of SSR based on motif types in C.parthenoxylon transcriptome Data type genomic File: oo_1000482.doc https://binary.pensoft.net/file/1000482 Mai-Phuong Pham, Cui Bei, Thi Tuyet Xuan Bui, Dinh Giap Vu, Syed Noor Muhammad Shah, Dinh Duy Vu

79628E78-59F9-5A86-A4E9-BED586C6730A 10.3897/BDJ.12.e123405.suppl2 Supplementary material 2 Table S2. Characterisation and polymorphism levels of 15 microsatellite loci in C.parthenoxylon Data type genomic File: oo_1023780.doc https://binary.pensoft.net/file/1023780 Mai-Phuong Pham, Cui Bei, Thi Tuyet Xuan Bui, Dinh Giap Vu, Syed Noor Muhammad Shah, Dinh Duy Vu

66E8B47A-183E-54B2-ADB5-EA2638C1E61B 10.3897/BDJ.12.e123405.suppl3 Supplementary material 3 Fig. S1 Distribution of contigs lengths resulting from de novo transcriptome assembly of C.parthenoxylon Data type images File: oo_1023779.png https://binary.pensoft.net/file/1023779 Mai-Phuong Pham, Cui Bei, Thi Tuyet Xuan Bui, Dinh Giap Vu, Syed Noor Muhammad Shah, Dinh Duy Vu

E06CF946-EB93-572A-A7FF-95CE50846558 10.3897/BDJ.12.e123405.suppl4 Supplementary material 4 Fig. S2 Distribution of transcripts lengths resulting from de novo transcriptome assembly of C.parthenoxylon Data type images File: oo_1023776.png https://binary.pensoft.net/file/1023776 Mai-Phuong Pham, Cui Bei, Thi Tuyet Xuan Bui, Dinh Giap Vu, Syed Noor Muhammad Shah, Dinh Duy Vu

89374DC7-571E-54DC-AE37-D087C785D0B2 10.3897/BDJ.12.e123405.suppl5 Supplementary material 5 Fig. S3 Distribution of unigenes lengths resulting from de novo transcriptome assembly of C.parthenoxylon Data type images File: oo_1000532.jpg https://binary.pensoft.net/file/1000532 Mai-Phuong Pham, Cui Bei, Thi Tuyet Xuan Bui, Dinh Giap Vu, Syed Noor Muhammad Shah, Dinh Duy Vu

1C3E20B0-43D6-55F7-9572-90B2A641BD21 10.3897/BDJ.12.e123405.suppl6 Supplementary material 6 Fig. S4 Distribution type of EST-SSRs of C.parthenoxylon Data type images File: oo_1023778.jpg https://binary.pensoft.net/file/1023778 Mai-Phuong Pham, Cui Bei, Thi Tuyet Xuan Bui, Dinh Giap Vu, Syed Noor Muhammad Shah, Dinh Duy Vu

## Figures and Tables

**Table 1.** Sampling location and genetic diversity within *C.parthenoxylon* populations at 15 SRR loci

**Population code**	**Information**	**Longitude (N) / Latitude (E)**	**N**	***Na***	***Ne***	***P***%	***H o***	***H e***	***Fis***	***F_is_IIM***	**P v a lue of bottleneck**	
											**TPM**	**SMM**
**DL**	Kon Chu Rang National Reserve, Gia Lai Province	14°31'13.5", 108°33'39.8"	40	4.13	2.91	93.33	0.49	0.58	0.14	0.027	ns	ns
**GL**	Chu Yang Sin National Park, Dak Lak Province	12°26'45.7", 108°18'20"	12	4.40	2.71	100.00	0.54	0.55	0.09	0.010	0.01	ns
**TH**	Xuan Lien National Reserve, Thanh Hoa Province	19°50'16.6", 105°13'45.7"	20	3.93	2.40	100.00	0.53	0.54	0.01	0.018	ns	ns
**QN**	Tay Giang District, Quang Nam Province	15°48'29", 107°19'8.6"	20	3.80	2.21	100.00	0.50	0.49	-0.03	0.017	ns	ns
**VP**	Tam Dao National Park, Tam Dao Province	21°25'15.8", 105°37'31.2"	19	2.40	1.85	80.00	0.71	0.40	-0.71	0.022	0.02	0.05
**HB**	Pa Co National Reserve, Hoa Binh Province	20°37'57", 105°26'19.9"	8	2.40	1.84	86.67	0.39	0.39	0.00	0.038	ns	ns
**PY**	Song Hinh District, Phu Yen Province	12°48'19.9", 109°00'9.8"	22	2.67	1.85	100.00	0.74	0.41	-0.64	0.015	ns	ns
**YT**	Yen Tu National Forests, Quang Ninh Provice	21°8'8.8", 106°45'24.2"	19	2.73	1.84	86.67	0.49	0.39	-0.19	0.018	ns	ns
**PT**	Xuan Son National Park, Phu Tho Province	21°10'8.3", 105°7'27.7"	19	3.13	2.00	100.00	0.69	0.45	-0.40	0.019	ns	ns
**Species level**					3.29	2.18	94.07	0.56	0.47	-0.19			
Note: N, population size; Na, mean number of alleles per locus; Ne, mean number of effective alleles; P %, percentage of polymorphic loci; Ho and He, mean observed and expected heterozygosities, respectively; Fis, inbreeding coefficient; F_is_IIM, corrected inbreeding coefficient for null alleles; *p < 0.05. ^∗∗^P < 0.01, *^∗∗∗^*P < 0.001; ns, not significant; *SMM (stepwise mutation model) and TPM (two-phased model of mutation)*.													

**Table 2.** Overview of *de novo* transcriptome assembly for *C.parthenoxylon*.

**Length range (bp)**	**Unigene**	**Contigs**	**Transcripts**
200-300	71,571 (44.61%)	3,167,763 (96.75%)	81,432 (30.96%)
300-500	44,960 (28.02%)	53,394 (1.63%)	58,136 (22.11%)
500-1000	25,658 (15.99%)	32,240 (0.98%)	47,630 (18.11%)
1000-2000	11,776 (7.34%)	14,348 (0.44%)	43,206 (16.43%)
> 2000	6,470 (4.03%)	6,526 (0.2%)	32,595 (12.39%)
Total Number	160,435	3,274,271	262,999
Total Length	88,071,463	257,547,156	237,355,325
N50 Length	711	110	1,682
Mean Length	548.95	78.66	902.49

**Table 3.** Functional annotation of *C.parthenoxylon* in different databases.

**Annotated database**	**Annotated_No.**	**Percentage (%)**	**300–1000 (bp)**	≥ **1000 (bp)**
COG	14,629	9.12	4,230	5,119
GO	25,352	15.80	8,776	7,345
KEGG	10,701	6.67	3,553	3,250
KOG	26,823	16.72	9,584	8,365
Pfam	29,514	18.39	10,044	11,310
Swissprot	25,202	42.35	9,431	9,098
Nr	49,383	30.78	18,276	13,831
All	50,691	31.59	18,704	13,879

**Table 4.** The distribution of EST-SSRs based on the number of repeat units.

**Number of repeat units**	**Mono**-	**Di**-	**Tri**-	**Tetra**-	**Penta**-	**Hexa**-	**Total**	**Percentage (%)**
5	0	0	1285	97	14	8	1404	10.93
6	0	871	609	18	3	7	1508	11.74
7	0	589	272	0	0	1	862	6.71
8	0	589	19	0	0	1	609	4.74
9	0	699	1	0	0	0	700	5.45
10	2041	455	0	0	0	0	2496	19.43
>10	5142	128	0	0	0	0	5270	41.01
Total	7183	3331	2186	115	17	17		100
Percentage (%)	55.90	25.92	17.01	0.90	0.13	0.13	100	

**Table 5.** Genetic parameters in 15 SSR loci for *C.parthenoxylon*.

**Primers**	***Na***	***Ne***	***Null allele***	***PIC***	***Ho***	***He***	***Fis***	***Fit***	***Fst***	***Nm***	***P*_HWE_**
**VDD01**	3.78	2.81	No	0.75	0.78	0.62	-0.29	0.03	0.22	0.89	ND
**VDD02**	3.78	2.50	No	0.77	0.72	0.59	-0.21	-0.16	0.04	5.68	***
**VDD03**	2.56	1.93	No	0.56	0.65	0.47	-0.37	-0.29	0.08	3.06	***
**VDD04**	3.56	2.36	No	0.42	0.66	0.57	-0.14	0.03	0.16	1.34	***
**VDD05**	3.33	1.98	No	0.61	0.61	0.47	-0.28	-0.16	0.11	2.07	***
**VDD06**	4.89	2.93	No	0.46	0.77	0.63	-0.27	-0.09	0.11	2.00	NS
**VDD07**	2.44	1.38	0.18	0.67	0.17	0.23	0.20	0.49	0.28	0.64	***
**VDD08**	3.33	2.05	0.12	0.30	0.32	0.36	0.11	0.30	0.21	0.95	***
**VDD09**	3.11	2.16	No	0.46	0.65	0.53	-0.24	-0.10	0.11	2.12	***
**VDD10**	2.89	1.94	No	0.52	0.63	0.46	-0.35	-0.22	0.11	1.95	**
**VDD11**	2.00	1.24	0.17	0.43	0.08	0.14	0.35	0.50	0.17	1.22	NS
**VDD12**	4.56	3.09	No	0.18	0.86	0.64	-0.37	-0.23	0.07	3.25	ND
**VDD13**	4.44	3.11	No	0.61	0.79	0.66	-0.24	-0.08	0.11	2.08	***
**VDD14**	2.33	1.43	0.09	0.71	0.18	0.21	0.00	0.31	0.22	0.90	***
**VDD15**	2.33	1.75	No	0.27	0.59	0.42	-0.38	-0.34	0.06	4.23	ND

Note: The number of alleles per locus (Na), the mean number of effective alleles (Ne), the average null allele frequency (Null allele), polymorphism information content (PIC), observed heterozygosities (Ho), expected heterozygosities (He), the fixation index (Fis), coefficient of total inbreeding (Fit), genetic differentiation index of Weir and Cockerham (1984) (Fst), gene flow (Nm) NS=not significant, ND=not determined *P<0.05, **P<0.01, ***P<0.001.

**Table 6.** Analysis of molecular variance in *C.parthenoxylon* from nine populations.

**Source of variation**	**Degree of freedom**	**Sum of squares**	**Variance components**	**Total variation (%)**	**P value**
**Amongst populations**	8	177.142	0.493	10	0.001
**Amongst individuals within populations**	170	504.179	0.000	0	
**Within individuals**	179	774.500	4.327	90	
**Total**	357	1455.821	4.820	100	

**Table 7.** Population pairwise Fst and significant values of the probability (*p-value* < 0.05).

	**GL**	DL	**TH**	**QN**	**VP**	**HB**	**PY**	**YT**	**PT**
**GL**	-	+	+	+	+	+	+	+	+
**DL**	0.07	-	+	+	+	+	+	+	+
**TH**	0.09	0.05	-	+	+	+	+	+	+
**QN**	0.09	0.06	0.04	-	+	+	+	+	+
**VP**	0.14	0.10	0.07	0.07	-	+	+	+	+
**HB**	0.13	0.05	0.05	0.06	0.08	-	+	+	+
**PY**	0.13	0.11	0.08	0.05	0.03	0.09	-	+	+
**YT**	0.14	0.08	0.07	0.06	0.03	0.06	0.04	-	+
**PT**	0.12	0.08	0.05	0.05	0.01	0.06	0.03	0.03	-
