# Supplementary material for: Characterisation of the Cinnamomumparthenoxylon (Jack) Meisn (Lauraceae) transcriptome using Illumina paired-end sequencing and EST-SSR markers development for population genetics
Source: Biodivers Data J. 2024 Jun 17;12:e123405. doi: 10.3897/BDJ.12.e123405 (PMC11196892; doi:10.3897/BDJ.12.e123405)
Supplement: Supplementary material 1 — Table S1. Frequency distribution of SSR based on motif types in C.parthenoxylon transcriptome [file bdj-12-e123405-s001.doc]

| **Table S1.** Frequency distribution of SSR based on motif types in *C. parthenoxylon* transcriptome | | | | | | | | | |
| --- | --- | --- | --- | --- | --- | --- | --- | --- | --- |
| Microsatellite motif | **Number of repeats** | | | | | | | **Total** | **Percentage (%)** |
|  | **5** | **6** | **7** | **8** | **9** | **10** | **>10** |  |  |
| A/T | - | - | - | - | - | 2024 | 5104 | 7128 | 55.475 |
| C/G | - | - | - | - | - | 17 | 38 | 55 | 0.428 |
| AC/GT | - | 115 | 69 | 45 | 27 | 20 | 22 | 298 | 2.319 |
| AG/CT | - | 617 | 432 | 437 | 543 | 343 | 84 | 2456 | 19.114 |
| AT/AT | - | 135 | 88 | 107 | 129 | 92 | 22 | 573 | 4.459 |
| CG/CG | - | 4 |  |  |  |  | 0 | 4 | 0.031 |
| AAC/GTT | 53 | 22 | 27 |  | 1 |  | 0 | 103 | 0.802 |
| AAG/CTT | 464 | 274 | 101 | 3 |  |  | 0 | 842 | 6.553 |
| AAT/ATT | 96 | 52 | 31 | 3 |  |  | 0 | 182 | 1.416 |
| ACC/GGT | 80 | 33 | 7 | 2 |  |  | 0 | 122 | 0.949 |
| ACG/CGT | 23 | 9 | 1 | 2 |  |  | 0 | 35 | 0.272 |
| ACT/AGT | 12 | 7 | 5 |  |  |  | 0 | 24 | 0.187 |
| AGC/CTG | 182 | 61 | 31 | 3 |  |  | 0 | 277 | 2.156 |
| AGG/CCT | 164 | 79 | 47 | 3 |  |  | 0 | 293 | 2.280 |
| ATC/ATG | 181 | 59 | 17 | 3 |  |  | 0 | 260 | 2.024 |
| CCG/CGG | 30 | 13 | 5 |  |  |  | 0 | 48 | 0.374 |
| AAAC/GTTT | 6 | 1 |  |  |  |  | 0 | 7 | 0.054 |
| AAAG/CTTT | 19 | 6 |  |  |  |  | 0 | 25 | 0.195 |
| AAAT/ATTT | 28 |  |  |  |  |  | 0 | 28 | 0.218 |
| AACC/GGTT | 2 | 1 |  |  |  |  | 0 | 3 | 0.023 |
| AAGG/CCTT | 2 |  |  |  |  |  | 0 | 2 | 0.016 |
| AATC/ATTG | 3 |  |  |  |  |  | 0 | 3 | 0.023 |
| AATG/ATTC | 6 |  |  |  |  |  | 0 | 6 | 0.047 |
| AATT/AATT | 1 |  |  |  |  |  | 0 | 1 | 0.008 |
| ACAG/CTGT | 1 |  |  |  |  |  | 0 | 1 | 0.008 |
| ACAT/ATGT | 4 | 2 |  |  |  |  | 0 | 6 | 0.047 |
| ACCG/CGGT | 1 |  |  |  |  |  | 0 | 1 | 0.008 |
| ACGC/CGTG | 1 |  |  |  |  |  | 0 | 1 | 0.008 |
| ACTC/AGTG | 1 |  |  |  |  |  | 0 | 1 | 0.008 |
| ACTG/AGTC | 1 |  |  |  |  |  | 0 | 1 | 0.008 |
| AGAT/ATCT | 10 | 2 |  |  |  |  | 0 | 12 | 0.093 |
| AGCC/CTGG |  | 2 |  |  |  |  | 0 | 2 | 0.016 |
| AGCG/CGCT | 4 |  |  |  |  |  | 0 | 4 | 0.031 |
| AGGC/CCTG |  | 1 |  |  |  |  | 0 | 1 | 0.008 |
| AGGG/CCCT | 1 | 1 |  |  |  |  | 0 | 2 | 0.016 |
| ATCC/ATGG | 3 | 2 |  |  |  |  | 0 | 5 | 0.039 |
| ATCG/ATCG | 3 |  |  |  |  |  | 0 | 3 | 0.023 |
| AAAAG/CTTTT | 2 |  | 1 |  |  |  | 0 | 2 | 0.016 |
| AAAAT/ATTTT | 1 |  | 2 |  |  |  | 0 | 1 | 0.008 |
| AAACT/AGTTT | 1 |  | 3 |  |  |  | 0 | 1 | 0.008 |
| AAAGG/CCTTT |  | 1 | 4 |  |  |  | 0 | 1 | 0.008 |
| AACAC/GTGTT | 1 |  | 5 |  |  |  | 0 | 1 | 0.008 |
| AACCC/GGGTT | 1 |  | 6 |  |  |  | 0 | 1 | 0.008 |
| AAGAG/CTCTT |  | 1 | 7 |  |  |  | 0 | 1 | 0.008 |
| AAGTG/ACTTC |  | 1 | 8 |  |  |  | 0 | 1 | 0.008 |
| AATAT/ATATT | 1 |  | 9 |  |  |  | 0 | 1 | 0.008 |
| AATCG/ATTCG | 1 |  | 10 |  |  |  | 0 | 1 | 0.008 |
| AATCT/AGATT | 1 |  | 11 |  |  |  | 0 | 1 | 0.008 |
| AGATC/ATCTG | 1 |  | 12 |  |  |  | 0 | 1 | 0.008 |
| ATCCC/ATGGG | 1 |  | 13 |  |  |  | 0 | 1 | 0.008 |
| ATCCG/ATCGG | 1 |  | 14 |  |  |  | 0 | 1 | 0.008 |
| ATCGC/ATGCG | 1 |  | 15 |  |  |  | 0 | 1 | 0.008 |
| ATGCC/ATGGC | 1 |  | 16 |  |  |  | 0 | 1 | 0.008 |
| AAAAAG/CTTTTT |  | 1 |  |  |  |  | 0 | 1 | 0.008 |
| AAACCC/GGGTTT | 1 |  |  |  |  |  | 0 | 1 | 0.008 |
| AAATCG/ATTTCG | 1 |  |  |  |  |  | 0 | 1 | 0.008 |
| AAATGG/ATTTCC |  | 1 |  |  |  |  | 0 | 1 | 0.008 |
| AACACC/GGTGTT | 1 |  |  |  |  |  | 0 | 1 | 0.008 |
| AACCTC/AGGTTG |  | 1 |  |  |  |  | 0 | 1 | 0.008 |
| AACTCC/AGTTGG | 1 |  |  |  |  |  | 0 | 1 | 0.008 |
| AAGACG/CGTCTT |  | 1 |  |  |  |  | 0 | 1 | 0.008 |
| AAGGAG/CCTTCT | 1 |  |  |  |  |  | 0 | 1 | 0.008 |
| AAGGTG/ACCTTC |  | 1 |  |  |  |  | 0 | 1 | 0.008 |
| AATTCC/AATTGG | 1 |  |  |  |  |  | 0 | 1 | 0.008 |
| ACCATG/ATGGTC |  |  | 1 |  |  |  | 0 | 1 | 0.008 |
| ACGAGG/CCTCGT |  | 1 |  |  |  |  | 0 | 1 | 0.008 |
| ACTCCG/AGTCGG |  | 1 |  |  |  |  | 0 | 1 | 0.008 |
| AGAGAT/ATCTCT |  |  |  | 1 |  |  | 0 | 1 | 0.008 |
| AGATGC/ATCTGC | 1 |  |  |  |  |  | 0 | 1 | 0.008 |
| AGCAGG/CCTGCT | 1 |  |  |  |  |  | 0 | 1 | 0.008 |
