# Supplementary material for: Characterisation of the Cinnamomumparthenoxylon (Jack) Meisn (Lauraceae) transcriptome using Illumina paired-end sequencing and EST-SSR markers development for population genetics
Source: Biodivers Data J. 2024 Jun 17;12:e123405. doi: 10.3897/BDJ.12.e123405 (PMC11196892; doi:10.3897/BDJ.12.e123405)
Supplement: Supplementary material 2 — Table S2. Characterisation and polymorphism levels of 15 microsatellite loci in C.parthenoxylon [file bdj-12-e123405-s002.doc]

| **Table S2.** Characterization and polymorphism levels of 15 microsatellite loci in *C. parthenoxylon* | | | | | |
| --- | --- | --- | --- | --- | --- |
| **Primers** | **Primer sequence (5'–3')** | **Repeat motif** | **Fragment size (bp)** | **Ta (**  **°C)** | **GenBank accession no** |
| **VDD01** | F: ACAGAAGTGAACATTCCCGC  R: TGCTGAAATGGGTGCTTGTA | (TCACGA)6 | 120-153 | 55 | OR536945 |
| **VDD02** | F: CATCCACGTCAACTCCATTG  R: CATAGCAAGCCTTCCGAGTC | (AGG)7 | 104-131 | 55 | OR536868 |
| **VDD03** | F: CGCGAGTCTGGGTAATAAGC  R: GAGAAAATGGTGCAGGCAAT | (CTT)8 | 130-230 | 55 | OR536874 |
| **VDD04** | F: CAAAGTTACGGGCATGCTTT  R: AGTCTCCAAACTCAAGGCCA | (CAG)7 | 246-258 | 55 | OR536886 |
| **VDD05** | F: TGCTGTGGCTGATACGAGTC  R: AGCACACGAACAACACAAGC | (ATCCG)5 | 230-260 | 55 | OR536940 |
| **VDD06** | F: GCAGCCAGAAGTGGAACATT  R: ATTTCCCAATTCCCACACAA | (GTC)7 | 143-176 | 55 | OR536854 |
| **VDD07** | F: ATTGTGGGATTGCCTACTGC  R: TCCATCTCTGCCTTTCAAGC | (AT)11 | 192-220 | 55 | OR536843 |
| **VDD08** | F: AACAGAATCCAGCCATGGAG  R: TGTTGGACTTGGGGGAGATA | (GGA)7 | 106-130 | 55 | OR536901 |
| **VDD09** | F: TGCAACCACCACAACTTTCT  R: ACTGCCCAATCAAGCACTCT | (ATA)7 | 124-151 | 55 | OR536877 |
| **VDD10** | F: AAGGGAGTGGAGACAAGGGT  R: CAATAGAACTGCACTCGGCA | (AGT)8 | 196-211 | 55 | OR536855 |
| **VDD11** | F: AAACTCACAAGCATCATCATCA  R: AGAGCTGGATTTGGGGTCTT | (ACCAC)9 | 211-233 | 55 | OR536941 |
| **VDD12** | F: TTGGATTGGAAGTCGGAGAC  R: TACGACCTCAACATGTCCCA | (GTAT)6 | 237-258 | 55 | OR536945 |
| **VDD13** | F: AAAGTCCAGCACAAACACCC  R: GGGTGCAGGAATAAGTTCCA | (ACT)7 | 113-131 | 55 | OR536933 |
| **VDD14** | F: ATCGAAATGGCGTATCGAAG  R: GAGAGAGGAATCGCGATGAG | (TCC)7 | 170-179 | 55 | OR536882 |
| **VDD15** | F: TGAGTGCACACCATTCTCTTCT  R: AACGTTTATTGGCTGTTGGC | (CTAGCC)5 | 181-196 | 55 | OR536946 |
