# Supplementary figures and images for: Characterisation of the Cinnamomumparthenoxylon (Jack) Meisn (Lauraceae) transcriptome using Illumina paired-end sequencing and EST-SSR markers development for population genetics
Source: Biodivers Data J. 2024 Jun 17;12:e123405. doi: 10.3897/BDJ.12.e123405 (PMC11196892; doi:10.3897/BDJ.12.e123405)

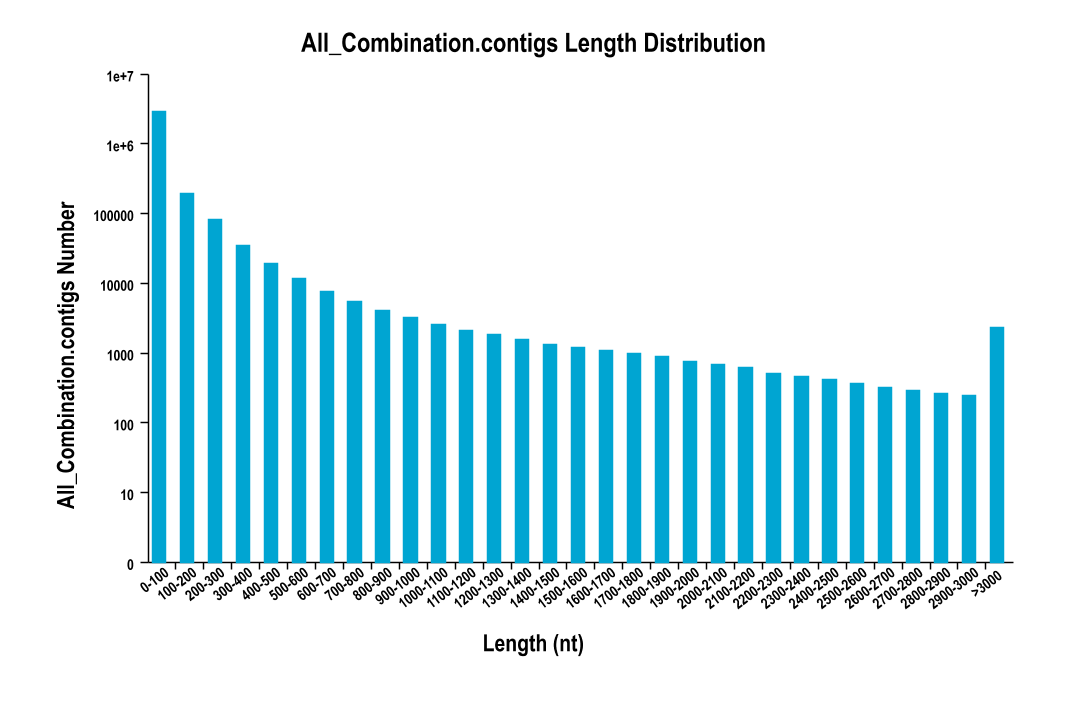

Supplement: Supplementary material 3 — Fig. S1 Distribution of contigs lengths resulting from de novo transcriptome assembly of C.parthenoxylon [file bdj-12-e123405-s003.png]

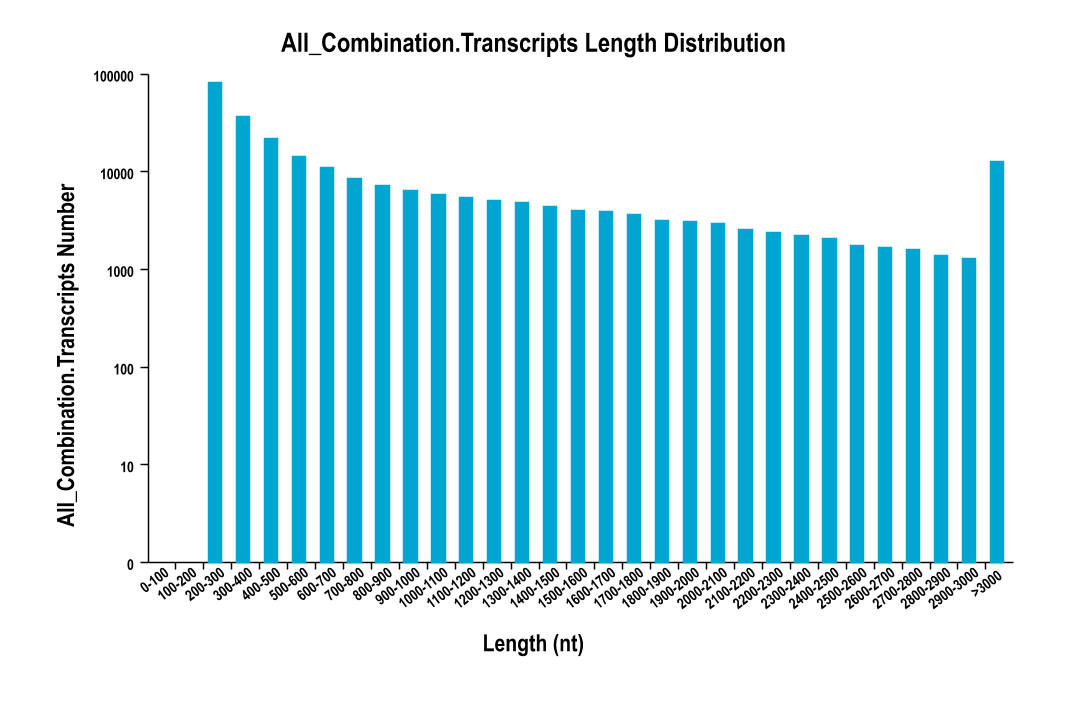

Supplement: Supplementary material 4 — Fig. S2 Distribution of transcripts lengths resulting from de novo transcriptome assembly of C.parthenoxylon [file bdj-12-e123405-s004.png]

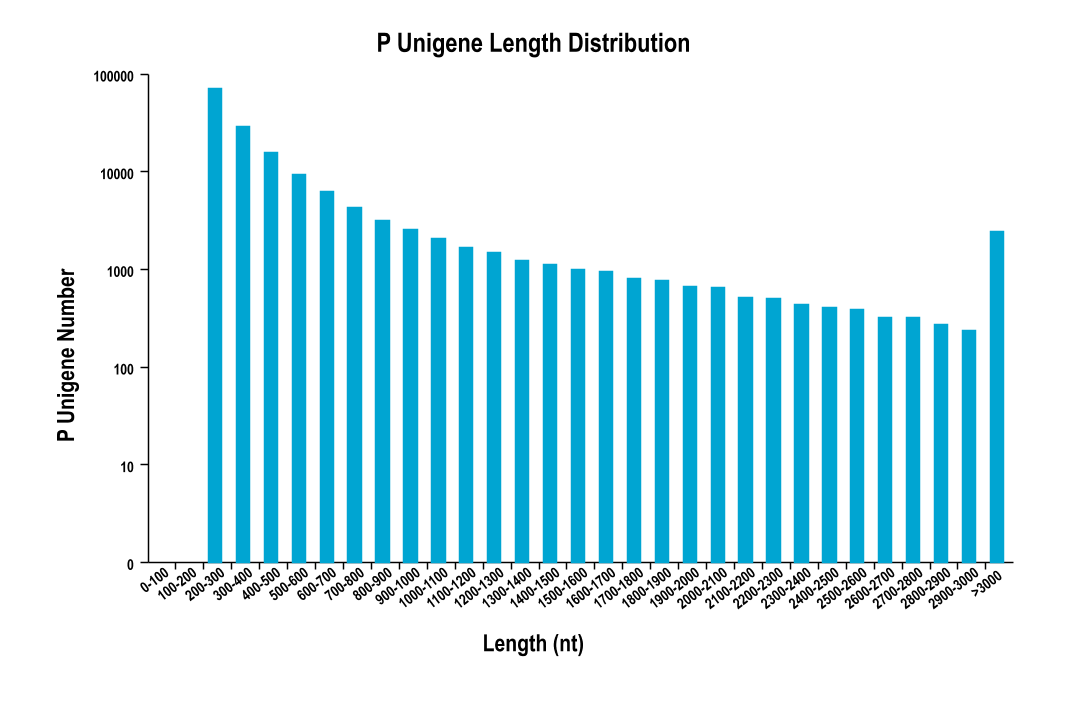

Supplement: Supplementary material 5 — Fig. S3 Distribution of unigenes lengths resulting from de novo transcriptome assembly of C.parthenoxylon [file bdj-12-e123405-s005.jpg]

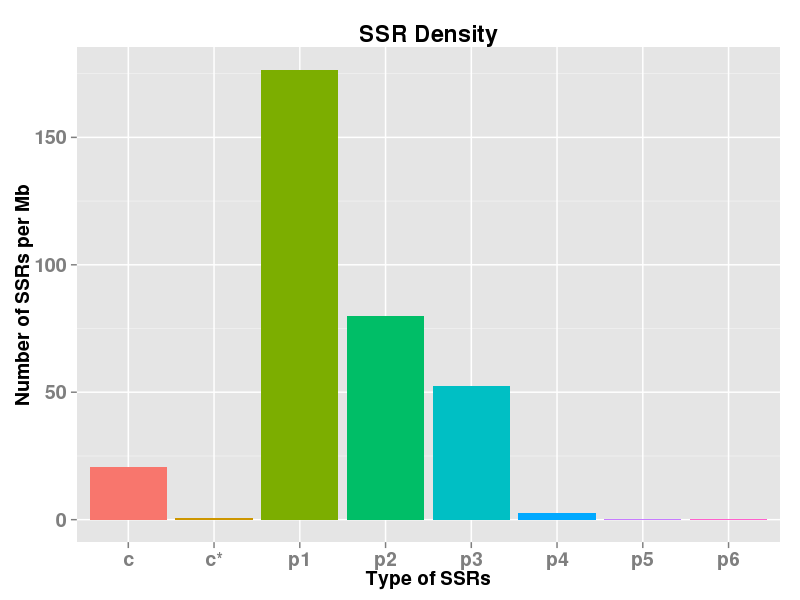

Supplement: Supplementary material 6 — Fig. S4 Distribution type of EST-SSRs of C.parthenoxylon [file bdj-12-e123405-s006.jpg]
